# The Current Roles of AI in Public Health Practice: Scoping Review

**DOI:** 10.2196/108250

**Published:** 2026-10-29

**Authors:** Sean Harrison, Claire Tatton, G J Melendez-Torres, Joelle Kirby, Sophie Robinson, Daniel Mutanda, Alisha Davies, Jessica M Armitage, Rabeea’h Waseem Aslam, Tom Arthur, Joht Singh Chandan, Ruth Garside, Jo Thompson Coon, Rhiannon Evans

**Affiliations:** 1 University of Exeter Medical School, University of Exeter, St Luke's Campus, Heavitree Road, Exeter, England, EX1 2LU, United Kingdom, 44 1392 661000; 2 Public Health Wales, Cardiff, Wales, United Kingdom; 3 Cardiff University, Cardiff, Wales, United Kingdom; 4 University of Birmingham, Birmingham, England, United Kingdom

**Keywords:** public health, AI, health protection, health improvement, scoping review

## Abstract

**Background:**

AI is a broad field encompassing various techniques, which include algorithms that learn from data to perform automated tasks without explicit human programming. Public health aims to protect and improve the health of communities and populations at the local, regional, national, and global levels, but it typically has limited resources. While AI may lead to improved efficiency or effectiveness of public health interventions, the adoption of AI interventions should be informed by evidence of effective use in practice.

**Objective:**

This review aims to summarize how AI-assisted interventions are currently used by public health professionals.

**Methods:**

We conducted a scoping review to identify the current uses of AI in public health. We searched MEDLINE (Ovid), Embase (Ovid), Web of Science (Core Collection), and Scopus for relevant reports published between January 1, 2020, and July 24, 2025. Two reviewers independently screened reports. Eligible reports were primary studies (experimental or observational), commentaries, and editorials that discussed how AI tools have been used in practice in public health contexts relevant to high-income countries, excluding outbreaks. Data were extracted by one reviewer and checked by a second.

**Results:**

We screened 5400 records and 155 full texts, including 8 reports. These reports discussed a variety of uses of AI tools across public health domains: 3 reports used chatbots in different settings, 1 report used machine learning to forecast health service demand, 2 reports used AI for symptom checking, and 2 reports used AI for evidence synthesis. While assessing the effectiveness of AI tools for public health was beyond the scope of this review, and most reports did not robustly evaluate effectiveness, the evidence for the effectiveness of AI tools was nonetheless mixed. One report described a chatbot that users found helpful, while another found a specific chatbot to be ineffective, highlighting its potential to undermine trust in the organization that developed it. Another report highlighted both benefits and drawbacks of a large language model used to communicate with socially isolated individuals. The main finding of this review was that even with a comprehensive search across multiple databases, there were few reports of how AI tools are being used in practice in public health contexts.

**Conclusions:**

The lack of evidence from public health practice, excluding outbreaks, makes it difficult to know which uses of AI, if any, would be useful to incorporate into practice in high-income countries. In the absence of evidence, new interventions, including those using AI, should be evaluated to ensure they are effective (and cost-effective). These evaluations should be widely and accessibly disseminated to create an evidence base that other public health teams could use to inform their own decisions around AI tools.

## Introduction

AI is a broad field encompassing various techniques, including machine learning, natural language processing, and generative AI [1]. AI learns from data to perform automated tasks without explicit human programming, using algorithms integrated into systems and tools [2-4]. While the theoretical groundwork for machine learning was laid in the 1980s, recent advancements in computing power and the development of large language models (LLMs) like OpenAI’s ChatGPT and Google’s Gemini have sparked renewed interest in AI [5]. These models, with more user-friendly interfaces and general-purpose applications, offer the potential to tackle a wide range of problems [6]. However, the rapid pace of development in AI generally, and in LLMs specifically, means new evidence assessing the effectiveness of interventions using AI tools is constantly required, as newer versions of these tools may have differential effectiveness.

Public health is an area wherein AI is increasingly being used to harness potential improvements in efficiency and effectiveness. This could be particularly useful, given the broad scope, important aims, and often limited resources of public health. In our recent reviews, we found several examples of chatbots, machine learning, and other AI-assisted interventions targeting alcohol consumption, smoking, and physical activity [7,8], and identified 44 potential uses of AI within public health [9].

Public health activities are often organized and described under 3 interconnected domains: health improvement, health protection, and health care public health [10]. Health improvement focuses on improving health and well-being and addressing inequalities through action on the wider determinants of health and by encouraging individual behavior change. Health protection focuses on the prevention and control of infectious diseases and emergency response. Health care public health focuses on ensuring that health care services are evidence-based, safe, and delivered effectively and equitably. AI tools could potentially be used across all these public health activities to improve services.

However, many reports on the potential effectiveness of AI interventions in public health use evidence from trials or from simulated or retrospective data. When moving into practice, the effectiveness of an intervention may be reduced, particularly in nonideal settings [11]. There may also be insurmountable barriers to adopting an intervention, for example, a lack of capacity, resources, or knowledge, particularly for technologically complex interventions [11]. Thus, it is important to evaluate how an intervention performs in routine use and in real-world contexts. Previous reviews have looked at AI in public health contexts [12,13], but they typically focus on evidence from trials or specific domains within public health, rather than real-world applications across public health more broadly.

The purpose of this scoping review is therefore to identify how AI is currently used in public health practice, that is, how AI-assisted interventions are currently used by public health professionals. In doing so, the review will provide a snapshot of the evidence base for using AI tools in public health practice, helping to inform which tools could be used to shape future public health practice. Scoping reviews are ideally placed to map the research landscape, detailing what has already been examined and hopefully illuminating promising areas.

## Methods

### Review Question

This scoping review systematically summarized evidence from primary studies, commentaries, and editorials to answer the following review question: how is AI currently used in public health?

This review formed part of a broader program of work evaluating the past, current, and potential uses of AI in public health [7-9]. The protocol was registered on Zenodo under the identifier 15971109 [14]. This review is reported using the PRISMA-ScR (Preferred Reporting Items for Systematic Reviews and Meta-Analyses extension for Scoping Reviews [15]; Checklist 1). All data produced in this review are contained in the manuscript and its supplementary information files. We did not use AI in any part of the review process or when writing this paper.

### Stakeholder Engagement

We conducted stakeholder engagement across our broader program of work across 2 phases of activity. The first phase involved protocol refinement and confirmation, with the aim of defining concepts related to AI and understanding comprehension of different models and platforms. The second phase gathered feedback on initial findings from the synthesis, with consideration of how public health systems may support diffusion and delivery of AI-based interventions.

We conducted consultation meetings with 2 UK-based communities of practice at each phase. One community comprised 9 practitioners from the National Institute for Health and Care Research (NIHR)–funded Health Determinants Research Collaborations (HDRCs), with experience of public health service delivery. HDRCs are local government-funded infrastructures intended to develop research capacity and capability to improve evidence-based decision-making. The second community, also comprising 9 people, included strategic directors with significant leadership roles in AI for public health across data science institutes, public health agencies, and national networks for local government organizations.

### Eligibility Criteria

We included primary studies (experimental or observational), commentaries, and editorials published or preprinted in English from January 1, 2020, that discussed how AI tools are currently used in public health contexts relevant to high-income countries (as defined by the World Bank [16]). We defined public health contexts as those where the primary aim was to improve public health, typically involving local and national government-funded public health organizations delivering interventions or making policy decisions. We excluded clinical settings, for instance, reports that only considered roles of AI using National Health Service (NHS) resources. Reports were only included if there was evidence that the AI tool had been used in public health practice; reports of potential uses of AI in public health contexts without evidence of having been used in practice were excluded, even if, for example, the AI tool was evaluated in a randomized controlled trial. Broadly, we considered use in practice to include public health professionals using AI tools for their work, as well as piloting, testing, or evaluating AI use, so long as these uses could have affected the public. For example, a report from a public health agency that had used ChatGPT to help write public-facing policies would be included, but a study comparing the readability of policies written with and without the help of ChatGPT (where the policies never became public-facing) would be excluded, as the AI tool was never used in practice. We defined AI broadly as algorithms integrated into systems and tools that can learn from data to be able to perform automated tasks without explicit human programming. Only public health contexts relevant to high-income countries were included, as this review was designed to be as relevant to the UK context as possible, and the public health challenges and resources are markedly different between high-income and other countries.

In the sole post hoc change to the protocol, we refined the eligibility criteria to exclude outbreaks, including local disease modeling and prediction, as AI use in outbreaks has been extensively researched [17]. This change was made before the search had been conducted, and our results are necessarily specific to AI use in public health practice, excluding outbreaks.

### Search Strategy

We used one AI search facet in the search strategy. This facet drew upon an unpublished filter created by members of the InterTASC (Technology Appraisal Support Collaboration) Information Specialists’ Sub-Group (ISSG), as well as filters hosted on the associated ISSG search filter resource [18]. We combined the AI search facet with broad public health terms, as well as those reflecting activities linked to the research question, such as policy making and surveillance. These terms were derived from iterative searching, with keywords and MeSH terms harvested from relevant papers and reference lists. Search strategies for all databases are presented in Multimedia Appendix 1.

### Information Sources

We conducted searches in 4 bibliographic databases: Medline (Ovid); Embase (Ovid, including preprints from medRxiv, bioRxiv, and SSRN); Web of Science (Core Collection); and Scopus. Searches were automatically deduplicated by DOI matching in Stata 19 (StataCorp LLC) [19]. Searches were conducted up to July 24, 2025.

### Selection of Sources of Evidence

Two reviewers (SH and CT) independently screened titles and abstracts in Covidence (Veritas Health Innovation Ltd) [20], and full texts in a standardized Microsoft Excel sheet. Titles and abstracts included by at least 1 reviewer on eligibility assessment progressed to full-text screening. Conflicts in assessment of full texts were resolved through discussion, with no necessity to involve a third party.

### Data Charting Process and Items

One reviewer (SH) completed all data extraction, which was checked by a second reviewer (CT). For each included report, we extracted information into standardized Microsoft Excel sheets about the report characteristics and methodology, the public health context, the type of AI and how it was used, and any outcomes associated with the performance of the AI intervention.

### Synthesis of Results

We summarized the evidence narratively, separately for each report. All data generated or analyzed during this review are included in this published paper and its supplementary information files.

### Ethical Considerations

Ethical approval for this review was not required. We conducted consultations with stakeholder groups in accordance with the ethical requirements stipulated by the organizations and research studies that recruited the participating members.

## Results

### Screening Results

Figure 1 shows the PRISMA diagrams for the flow of references. See Multimedia Appendix 2 for full-text screening results for reports and full data extractions for included reports.

**Figure 1.**
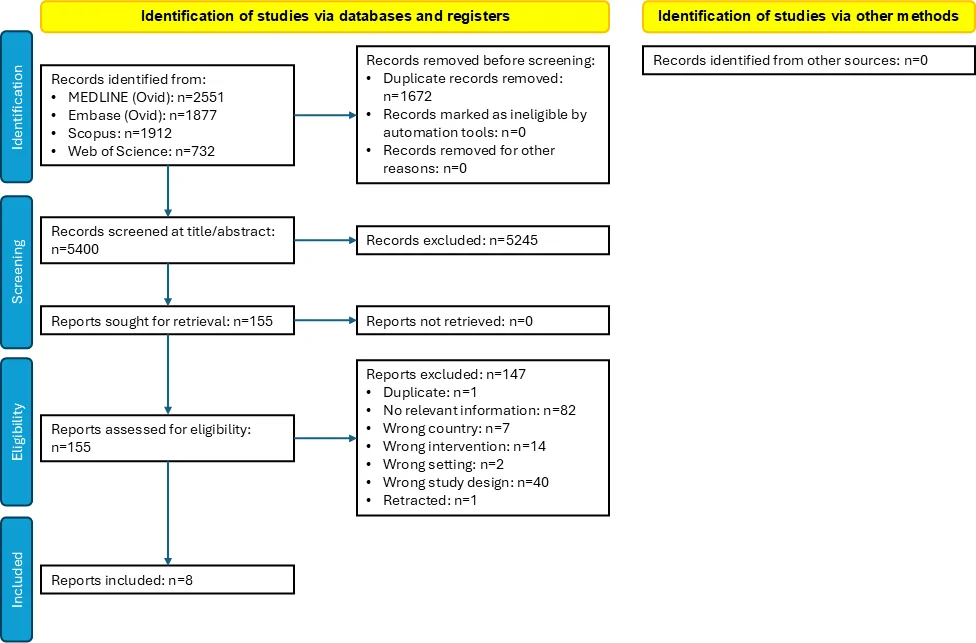
PRISMA (Preferred Reporting Items for Systematic Reviews and Meta-Analyses) diagram.

We screened 5400 titles and abstracts and 155 full texts, ultimately including 8 reports [21-28] that discussed AI tools used in public health practice. A total of 4 reports involved health improvement [21-24], 4 involved health care public health [23-26], and 5 involved health protection [22,24,26-28]; 4 of these reports involved multiple public health contexts [22-24,26] (Figure 2).

**Figure 2.**
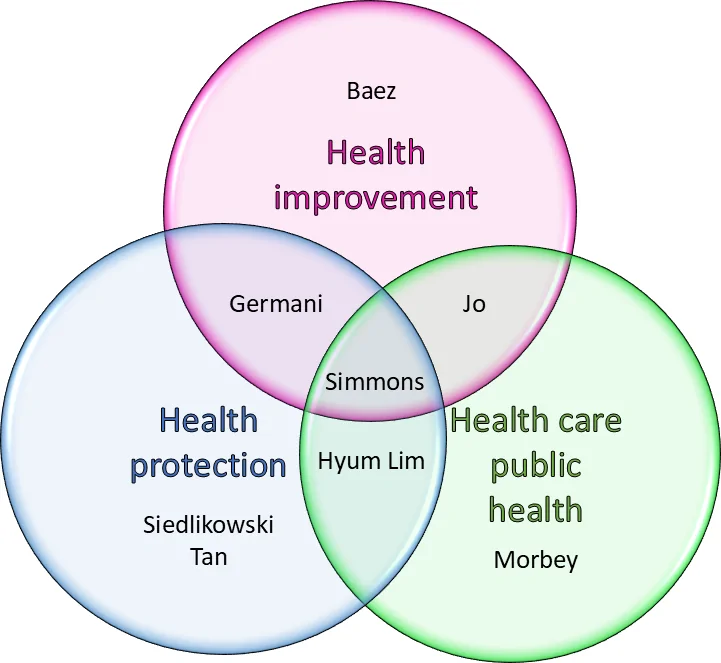
Public health contexts of included reports [21-28].

A total of 2 reports were from the United Kingdom [24,25], 2 from Canada [26,27], and 1 each from Singapore [28], South Korea [23], the United States [21], and worldwide [22]. A total of 5 reports assessed the accuracy of AI tools [24-28], 2 reports conducted interviews to qualitatively assess the utility of AI tools [21,23], and 1 report used structured interactions to assess the conversational and informational performance of an AI tool [22].

Table 1 provides an overview of the AI tools identified, while narrative summaries of included reports are provided below.

**Table 1.** Report characteristics.

Reference	Country, report dates, and participants	Report type and public health contexts	Outcome measurements	Intervention description
Baez et al [21], 2025	United States June to July, 2022 19 participants interviewed	Qualitative Health improvement	Open coding of interview recordings	Intervention: WIC^a^ chatbot (dialog-based): Maya Intervention description: The chatbot helped clients to search for local WIC offices and approved stores, report a lost or stolen card, and upload documentation and answered client questions.
Germani et al [22], 2025	Worldwide 2024 or 2025 no participants	Structured interaction Health improvement and health protection	Inductive, open, and deductive coding of S.A.R.A.H’s responses to ChatGPT prompts and responses	Intervention: WHO^b^ chatbot (generative AI): S.A.R.A.H Intervention description: the chatbot provided accessible and reliable health information while combating misinformation.
Hyun Lim et al [26], 2024	Canada (PHAC^c^) 2020 to 2023 No participants	Accuracy Health protection and health care public health	Correct screens, validated by human screening	Intervention: Evidence review software (natural language processing): DistillerSR Artificial Intelligence System (DAISY) Intervention description: To screen citations based on a prespecified eligibility criteria.
Jo et al [23], 2025	South Korea 2023 21 public agency workers	Qualitative Health improvement and health care public health	Open coding of interview recordings	Intervention: CareCall, a phone call chatbot (LLM^d^): HyperCLOVA Intervention description**:** CareCall mimicked the conversational style of a social worker to check on care recipients. The LLM accessed long-term memory to personalize open-ended conversations, including by asking follow-up questions. Public health agency workers were notified of potential concerns.
Morbey et al [25], 2025	United Kingdom (UKHSA^e^) 2022 to 2023 no participants	Accuracy Health care public health	Peak intensity and timing errors	Intervention: Short-term forecasting models for syndromic surveillance (machine learning): 2 machine-learning pipelines, to: (1) select and train forecast models, and (2) create daily forecasts Intervention description**:** machine learning for forecasting RSV^f^ from NHS^g^ 111 calls for “cough” and emergency department attendance for “acute bronchitis,” restricted to children aged 5 years and younger, with the aim of forecasting peak health care demand in the short-term. The random forest learning method with seasonality modeled by Fourier transformations was ultimately selected from the 252 trained models as it had the least error. This model was used to produce 1 to 28 day ahead forecasts using latest available UKHSA data (winter of 2022 to 2023).
Siedlikowski et al [27], 2021	Canada Launched March 2020 no participants	Accuracy Health protection	Overall accuracy and out-of-distribution accuracy	Intervention: COVID-19 chatbot (machine learning): Chloe Intervention description**:** Chloe was designed to help users self-assess COVID-19 symptoms and to answer common questions in both English and French about COVID-19, which were clustered using unsupervised machine learning. COVID-19 information used to train the chatbot was scraped from vetted online information sources. A BERT^h^ model was used to detect and remove out-of-distribution questions, which were not answerable with available information sources used by the chatbot. Users could freely ask questions, which the chatbot parsed through natural language understanding.
Simmons et al [24], 2025	United Kingdom (UKHSA) 2022 to 2025 Deduklick: 8 reviews; Rayyan and EPPI-Reviewer: 1 review	Accuracy Health improvement, health protection, and health care public health	Deduklick: comparison with Leeds Method and general feedback; Rayyan and EPPI-Reviewer: how many records had to be screened to find all records in the prioritized list	Intervention: Deduklick (AI, not specified); Rayyan Prediction Classifier (Support Vector Machine classifier); EPPI-Reviewer Priority Screening (machine learning) Intervention description**:** Deduklick: removing duplicate records in reviews (no further details); Rayyan and EPPI-Reviewer: prioritize records when screening based on previous inclusion decisions.
Tan et al [28], 2024	Singapore 2022 200 images	Accuracy Health protection	Sensitivity and specificity of symptomatic Mpox infection	Intervention: HeHealth app (modified convolutional neural network) Intervention description: To examine images of penises, based on training from 1000 images of penises with mpox positive rashes and 1000 images of penises without mpox rashes. Additional information about the geographical prevalence of mpox and latest positive patient statistical data was used to predict the likelihood of mpox.

a WIC: Special Supplemental Nutrition Program for Women, Infants, and Children.

b WHO: World Health Organization.

c PHAC: Public Health Agency of Canada.

d LLM: large language model.

e UKHSA: United Kingdom Health Security Agency.

f RSV: respiratory syncytial virus.

g NHS: National Health Service.

h BERT: bidirectional encoder representations from transformers.

### The Current Roles of AI in Public Health

Baez et al [21] interviewed 19 mothers from the United States in 2022 to determine whether a chatbot on the Texas Special Supplemental Nutrition Program for Women, Infants, and Children (WIC) website was useful, and how it could be improved. The chatbot was called Maya, and helped clients to search for local WIC offices and approved stores, report a lost or stolen card, and upload documentation, while answering client questions. Participants stated that Maya helped them navigate and find information on the Texas WIC website, and that Maya was more efficient for obtaining information (particularly about their benefits) than other practices, such as by visiting a clinic, calling a hotline, or emailing. As of January 2026, Maya is still active on the Texas WIC website [29].

Germani et al [22] assessed the conversational and informational performance of the World Health Organization (WHO) chatbot, S.A.R.A.H, by presenting it with 10 statements generated by GPT-3.5-turbo that contained health-related misinformation. S.A.R.A.H was developed to provide accessible and reliable health information and combat misinformation. The authors noted that some of S.A.R.A.H’s responses failed to directly address user concerns (redirecting the conversation instead), lacked conversational skills associated with empathy, and failed to provide relevant evidence-based information to debunk misinformation. However, the authors also noted that one of S.A.R.A.H’s responses acknowledged user concerns, suggested actional points, and showed artificial empathy. Overall, the authors concluded that S.A.R.A.H had “all the ingredients to serve not only as a useless tool in the quest to improve the information ecosystem and reduce the dangers infodemics pose to public health but also as a potential threat to erode trust in the WHO itself” [22]. As of January 2026, S.A.R.A.H is no longer active on the WHO website [30].

Hyum Lim et al [26], within the Public Health Agency of Canada, used the DistillerSR Artificial Intelligence System (DAISY) to assist in screening of reports and compared the accuracy of screening for relevant COVID-19 citations to that of human reviewers between 2020 and 2023. DAISY uses natural language processing and human-coded training data to automatically screen the titles and abstracts of citations. DAISY correctly classified 98% of the 21,729 citations that had undergone human review and found relevant citations missed by human reviewers (around 3% of included citations). As of January 2026, DistillerSR continues to use AI classifiers, although potentially not DAISY specifically [31].

Jo et al [23] interviewed 21 public agency workers in South Korea in 2023 to determine their expectations of, and experiences with, CareCall, an LLM-based chatbot that spoke with socially isolated people through phone calls. CareCall is being used in South Korea to supplement the role of public agency workers in monitoring socially isolated people, including their health. The chatbot mimics the conversational style of a social worker to check on care recipients and accesses long-term memory to personalize open-ended conversations, including by asking follow-up questions. Public health agency workers were notified of potential concerns, which could then be followed up on. Overall, adoption of CareCall seems to have been viewed positively by the workers interviewed. They felt that CareCall expanded public reach and allowed for more frequent monitoring but often without the necessary resources at implementation, which increased and diversified their workloads. Unlike before, there was no need for dedicated devices (such as sensor systems and smart speakers) because CareCall uses existing phone lines. In this regard, CareCall was therefore cheaper and reduced barriers to adoption. Different (additional) care needs were also communicated, allowing workers to provide necessary support that may otherwise have been missed. However, some workers felt that some participants who did not answer calls from CareCall did so because they did not like talking to an AI chatbot, and this added to workloads by necessitating callbacks to check that participants were safe.

Morbey et al [25], within the UK Health Security Agency (UKHSA), created and piloted pipelines to select and use machine learning models to forecast health care service demand in the UK in 2022 and 2023. Specifically, the machine learning models forecast the peak timing and intensity of NHS 111 calls for “cough” and emergency department attendance for “acute bronchitis” over 28 days, restricted to children aged 5 years and younger. Seven machine learning methods were trained on data from before October 2022 (80% training and 20% validation), with 4 options for seasonality, a secular trend, quadratic terms for current activity, and a 3-day moving average, for a total of 252 alternative model specifications, each trained to forecast 1-28 days ahead inclusive. The model with the smallest weighted combination of intensity error (difference between the predicted and observed peak demand) and timing error (difference between the predicted and observed peak day) was selected and retrained on all available data, and historical errors were incorporated into forecast uncertainty. The random forest learning method with seasonality modeled by Fourier transformations was ultimately selected. This model was then used to produce 1 to 28-day-ahead forecasts using the latest available UKHSA data (winter of 2022 to 2023), which were compared to the observed peaks in NHS 111 calls and emergency department attendances. As there was an unprecedented demand for NHS 111 calls for cough, the forecasts could not predict the peak demand until more data had been acquired, though the forecasts were able to correctly predict the peak emergency department acute bronchiolitis attendance.

Siedlikowski et al [27] discussed the development of Chloe, a chatbot released in March 2020, which was designed to help users self-assess COVID-19 symptoms and to answer common questions in both English and French about COVID-19. The COVID-19 information used to train the chatbot was scraped from vetted online information sources. A bidirectional encoder representations from transformers model was used to detect and remove out-of-distribution questions that were not answerable with the information sources available to the chatbot. Questions themselves were clustered using unsupervised machine learning. Users could freely ask questions, which the chatbot parsed using natural language understanding. The overall accuracy of Chloe was 81.8% in English and 71.3% in French, and the out-of-distribution accuracy was 90.5% in English and 70.6% in French. As of January 2026, Chloe is no longer active on its website [32].

Simmons et al [24], within the UKHSA, tested and subsequently adopted 3 different AI-assisted interventions to help with conduct evidence reviews in the United Kingdom between 2022 and 2025. They compared Deduklick (Risklick), software that uses AI to identify and remove duplicate records before screening, with manual deduplication in EndNote (Clarivate; using the Leeds Method) across 8 reviews, concluding that although Deduklick removed slightly fewer references, it saved considerable time and was adopted for use. They subsequently used both Rayyan’s prediction classifier (a support vector machine classifier) and EPPI-Reviewer’s Priority Screening (machine learning) to conduct priority screening in one review each. For Rayyan, all eventually included records were in the prioritized list after 32% of records had been screened, and for EPPI-Reviewer, 99% of eventually included records were in the prioritized list after 30% of records had been screened. Both tools have been adopted for use.

Tan et al [28] developed and tested an AI-assisted app that detected the presence of symptomatic mpox in images of penises in 2022. The app used a modified convolutional neural network trained on 1000 images of penises with mpox rashes and 1000 images of penises without mpox rashes, and additional information about the geographical prevalence of mpox and the latest positive patient statistical data, to predict the likelihood of symptomatic mpox in new images of penises. The authors stated that the app had a sensitivity of 90% and specificity of 87%, and that between June and October 2022, the app had analyzed 21,000 images of penises for mpox. The app is also used to predict the likelihood of other symptomatic sexually transmitted infections. However, as of October 2026, the app is no longer available, and the website has been shut down (though an archived version of the app’s web page is available [33]).

## Discussion

### Principal Findings

This scoping review summarized evidence on the current use of AI in public health relevant to high-income countries, excluding outbreaks. We identified a total of 8 reports [21-28] that discussed current uses of AI across all 3 public health domains. Uses of AI included using AI tools for symptom checking and for conducting evidence reviews for public health; using AI tools to address health concerns and misinformation and to connect individuals to services and support; and using machine learning to forecast health service demand.

While assessing the effectiveness of AI tools was beyond the scope of this review, the evidence from included reports was mixed. For example, while the Maya chatbot seemed helpful to participants [21], the S.A.R.A.H chatbot was considered a “potential threat to erode trust in the WHO” [22], and CareCall had both advantages and disadvantages for public agency workers and wasn’t evaluated by those at whom it was targeted [23]. Equally, in our reviews of the effectiveness of public health AI-assisted interventions for alcohol use, smoking, and physical inactivity, there was ultimately little evidence to suggest that AI-assisted interventions were any more or less effective than non-AI-assisted interventions [7,8].

All reports included in this review were authored by larger public health organizations, for example, national and global public health bodies, which may be more likely than smaller and more local public health teams to possess the infrastructure and capabilities to use and evaluate AI technologies. Although we identified only a small number of included reports, and any inferences drawn are therefore speculative, these authorship imbalances may also reflect varying levels of organizational readiness to use AI tools, relating to divergences in workforce capacity, training, infrastructure, equipment, and resources. If so, this variability may perpetuate differences in how AI is used and evaluated across all aspects of public health, potentially leading to inequities if AI tools are more effective or efficient than alternatives—risks of the potential use of AI in public health identified in our linked review [9]. However, the observed imbalances may also simply reflect differential publishing practices between larger and smaller public health organizations: smaller public health teams may be using AI tools but not publishing their experiences in academic journals. Indeed, our PPI groups noted that larger organizations may be slower to adopt AI due to more rigid governance systems than those of smaller organizations, which may be more flexible.

The rationale for identifying current uses of AI in this review was to determine how much real-world evidence exists and how easily accessible AI tools are in public health practice. Using a comprehensive search across 4 databases, we were able to identify only 8 reports [21-28]. On the other hand, in our review of the potential roles of AI in public health, we identified 67 reports covering 44 potential uses of AI across 8 public health contexts [9]. This may mean that there are plenty of potential uses of AI but little accessible evidence supporting those uses of AI in current practice.

The limitations in research evidence are important: indeed, the effectiveness of interventions tends to reduce when moving into practice, and unintended consequences may also become apparent. For example, the CareCall AI expanded public reach to socially isolated people, but this also increased the workload of public agency workers, as their caseload increased and some people did not want to communicate with AI [23]. In our review of the potential roles of AI in public health contexts, we identified a large number of risks, including the perpetuation or reinforcement of health inequalities and discrimination, concerns around transparency, data management, privacy, security, and confidentiality, and ethical, legal, and regulatory concerns, all of which may contribute to unintended and unpredictable consequences of AI adoption within public health [9].

Without underlying evidence of effectiveness in practice, and the numerous and critical risks, it would seem extremely difficult for public health teams to know which uses of AI, if any, would be useful to adopt into practice.

### Stakeholder Consultations

Stakeholders recognized the potential of AI-assistance in delivering behavior change interventions but highlighted a number of key knowledge gaps that need to be attended to moving forward. First, stakeholders noted limited reporting of the extent to which interventions were coproduced with the target audience, which raises important considerations of how coproduction methods can be optimally used in AI-based research. Second, stakeholders discussed the need for participants to have readiness and motivation to engage with AI and noted skill development may be required. Third, stakeholders queried the strength of implementation science in this area. For example, they considered if apps would be hosted by developers or local authorities, and what resources and capabilities would local public health teams require to support AI-assisted approaches. Fourth, stakeholders commented on the methodological limitations of evaluation, and whether new appraisal tools would need to be developed to account for the specific challenges and limitations associated with AI. Specifically, there was discussion of whether AI approaches have the potential to exacerbate health inequities and cause unintended harms, which have not yet been fully theorized or translated into appraisal criteria.

### Limitations of This Review

Despite the comprehensive search strategies, it is possible that relevant reports may have been missed from searches. Equally, despite duplication of screening, relevant reports may have been missed during screening, and relevant information may have been missed during data extraction of included reports. However, the purpose of this scoping review was only to determine how much real-world evidence exists and is easily accessible for using AI tools in public health: if we missed relevant evidence because it was not accessible, then public health teams may also miss that evidence when searching for the effectiveness of AI tools in public health.

Additionally, we focused solely on AI tools used within public health. These tools, or entirely different AI tools that could or have been used within public health but were not included in this review, may have evidence of use in other fields. However, the utility of tools may differ across fields, in a similar way to how effectiveness of interventions in trials may differ from effectiveness in practice.

Finally, defining and identifying both public health and which interventions are AI-assisted was challenging, as the definitions for both public health and AI are broad, and some reports provide little or no information about the AI aspect of AI-assisted interventions. We aimed to avoid missing relevant reports by taking a sensitive approach to screening and using broad inclusion criteria.

### Implications for Research and Practice

When considering adopting an AI intervention, public health professionals should carefully consider the potential effectiveness and cost-effectiveness alongside any potential risks of harm, even for nonclinical interventions [34]. This review identified some current uses of AI tools that look beneficial (the WIC chatbot, some uses in evidence synthesis), though the description of the S.A.R.A.H chatbot as both ineffective and a “potential threat to erode trust in the WHO” provides a stark warning of the risks of harm.

One key finding in this review is that while there are a multitude of potential uses of AI tools in public health, there is limited evidence for their use in practice. In the absence of evidence, new interventions, including those using AI, should ideally be evaluated by public health teams to ensure they are effective (and cost-effective). These evaluations should be widely and accessibly disseminated to create an evidence base that other public health teams could use to inform their own decisions around AI tools. This poses the opportunity and need for researchers and practitioners to work more closely to share skills and resources.

### Conclusions

This scoping review summarized evidence for the current uses of AI in public health relevant to high-income countries, excluding outbreaks. Across 3 public health contexts, we identified only 8 reports [21-28] of current uses of AI, which ultimately had mixed evidence of effectiveness. This lack of evidence from public health practice makes it difficult to know which uses of AI, if any, would be useful to adopt. In the absence of evidence, new interventions, including those using AI, should ideally be evaluated to ensure they are effective (and cost-effective). These evaluations should be widely and accessibly disseminated to create an evidence base that other public health teams could use to inform their own decisions around AI tools.

## Supplementary material

10.2196/108250 Multimedia Appendix 1 Search strategies.

10.2196/108250 Multimedia Appendix 2 Full text screening results and data extraction.

10.2196/108250 Checklist 1 PRISMA-ScR checklist.

## Abbreviations

DAISY: DistillerSR Artificial Intelligence System
HDRC: Health Determinants Research Collaboration
ISSG: InterTASC Information Specialists’ Sub-Group
LLM: large language model
NHS: National Health Service
NIHR: National Institute for Health and Care Research
PRISMA-ScR: Preferred Reporting Items for Systematic Reviews and Meta-Analyses extension for Scoping Reviews
TASC: Technology Appraisal Support Collaboration
UKHSA: UK Health Security Agency
WHO: World Health Organization
WIC: Special Supplemental Nutrition Program for Women, Infants, and Children
