# Supplementary material for: The Current Roles of AI in Public Health Practice: Scoping Review
Source: J Med Internet Res. 2026 Oct 29;28:e108250. doi: 10.2196/108250 (PMC13718742; doi:10.2196/108250)
Supplement: Supplementary file 1 [file jmir-v28-e108250-s001.docx]

# Search strategies

**Ovid MEDLINE(R) ALL (1946 to 28 July 2025)**

| **#** | **Query** | **Results** |
| --- | --- | --- |
| 1 | exp artificial intelligence/ | 244,919 |
| 2 | exp Machine Learning/ | 99,064 |
| 3 | (AI or AI4H).ti,ab. | 77,545 |
| 4 | ("comput* intelligence" or "comput* reasoning" or "machine intelligence" or "artificial intelligence" or "natural language processing" or llm*1 or "large language model*" or "language learning model*" or "classification algorithm*" or "computer heuristic*" or "convolutional network*" or "expert system*" or "recommender system*" or "decision support system*" or "decision tree" or "deep learning" or "data science" or "feature detection" or "generative pre-trained transformer" or "generative pretrained transformer" or "learning algorithm*" or "machine learning" or "neural net*" or "reinforcement learning" or "*supervised learning" or "intelligent agent*" or conversational agent* or chatbot*).ti,ab,kf. | 405,594 |
| 5 | ("boltzmann machine*" or "long short-term memory" or "gated recurrent unit*" or "rectified linear unit*" or autoencoder or "auto-encoder" or backpropagation or "multilayer perceptron" or "multi-layer perceptron" or convnet or "convolutional learning").ti,ab,kf. | 21,909 |
| 6 | ("Bing chat" or ChatGPT* or "Chat GPT" or "Google* Bard" or "Google* Gemini" or "IBM Watson" or "Microsoft* Bing" or Copilot or OpenAI or "Open AI" or PathAI or "Path AI" or DALL-E or invideo or Claude or Deepseek).ti,ab,kf. | 9,400 |
| 7 | 1 or 2 or 3 or 4 or 5 or 6 | 530,486 |
| 8 | exp *Public Health/ | 2,226,117 |
| 9 | public health/ | 101,459 |
| 10 | public health.ti,kf. | 141,566 |
| 11 | public health.ab. /freq=2 | 57,245 |
| 12 | exp Population Health/ | 41,451 |
| 13 | exp Primary Prevention/ | 196,010 |
| 14 | ((local or regional) adj (government* or authori* or council*)).ti,ab. | 14,284 |
| 15 | exp *Health Promotion/ | 58,542 |
| 16 | 8 or 9 or 10 or 11 or 12 or 13 or 14 or 15 | 2,541,752 |
| 17 | (evaluat* or decide* or decision* or case stud*).ti. | 892,126 |
| 18 | ("policy maker*" or policymaker* or "policy making" or policymaking).ti,kf. | 3,715 |
| 19 | (policy or policies).ab. /freq=3 | 47,716 |
| 20 | Policy Making/ | 18,572 |
| 21 | Public Policy/ | 34,340 |
| 22 | 17 or 18 or 19 or 20 or 21 | 979,775 |
| 23 | exp Population Surveillance/ | 75,719 |
| 24 | (infodemiology or infoveillance).ti,kf. | 838 |
| 25 | (misinformation or disinformation).ti. | 2,126 |
| 26 | Infodemiology/ | 85 |
| 27 | exp *Disease Outbreaks/sn [Statistics & Numerical Data] | 7,023 |
| 28 | Disease Outbreaks/pc [Prevention & Control] | 17,869 |
| 29 | Foodborne Diseases/pc [Prevention & Control] | 2,696 |
| 30 | *Foodborne Diseases/ep [Epidemiology] | 2,419 |
| 31 | ((prevent* or monitor* or control or surveillance or model?ing or forecast* or predict*) adj2 (outbreak* or pandemic* or epidemic*)).ti. | 3,053 |
| 32 | Health Policy/ | 75,559 |
| 33 | 23 or 24 or 26 or 27 or 28 or 29 or 30 or 31 or 32 | 177,234 |
| 34 | 22 or 33 | 1,135,565 |
| 35 | 7 and 16 and 34 | 3,863 |
| 36 | limit 35 to yr="2020 -Current" | 1,706 |
| 37 | Public health.ti,kf. | 141,566 |
| 38 | *artificial intelligence/ or *large language model/ | 35,019 |
| 39 | (artificial intelligence or AI or llm* or machine learning or deep learning or expert system* or chatbot* or conversational agent*).ti,kf. | 198,921 |
| 40 | 38 or 39 | 213,286 |
| 41 | 37 and 40 | 1,044 |
| 42 | limit 41 to yr="2020 -Current" | 973 |
| 43 | 36 or 42 | 2,551 |

**Embase (1974 to 24 July 2025)**

| **#** | **Query** | **Results** |
| --- | --- | --- |
| 1 | exp artificial intelligence/ | 151,235 |
| 2 | exp machine learning/ | 613,394 |
| 3 | (AI or AI4H).ti,ab. | 104,073 |
| 4 | ("comput* intelligence" or "comput* reasoning" or "machine intelligence" or "artificial intelligence" or "natural language processing" or llm*1 or "large language model*" or "language learning model*" or "classification algorithm*" or "computer heuristic*" or "convolutional network*" or "expert system*" or "recommender system*" or "decision support system*" or "decision tree" or "deep learning" or "data science" or "feature detection" or "generative pre-trained transformer" or "generative pretrained transformer" or "learning algorithm*" or "machine learning" or "neural net*" or "reinforcement learning" or "*supervised learning" or "intelligent agent*" or conversational agent* or chatbot*).ti,ab,kf. | 474,733 |
| 5 | ("boltzmann machine*" or "long short-term memory" or "gated recurrent unit*" or "rectified linear unit*" or autoencoder or "auto-encoder" or backpropagation or "multilayer perceptron" or "multi-layer perceptron" or convnet or "convolutional learning").ti,ab,kf. | 23,452 |
| 6 | ("Bing chat" or ChatGPT* or "Chat GPT" or "Google* Bard" or "Google* Gemini" or "IBM Watson" or "Microsoft* Bing" or Copilot or OpenAI or "Open AI" or PathAI or "Path AI" or DALL-E or invideo or Claude or Deepseek).ti,ab,kf. | 10,407 |
| 7 | 1 or 2 or 3 or 4 or 5 or 6 | 854,474 |
| 8 | exp *public health/ | 78,293 |
| 9 | preventative health service/ | 1 |
| 10 | exp *prevention/ | 613,112 |
| 11 | primary prevention/ | 51,449 |
| 12 | public health.ti,kf. | 111,591 |
| 13 | public health.ab. /freq=2 | 67,096 |
| 14 | exp population health/ | 8,186 |
| 15 | ((local or regional) adj (government* or authori* or council*)).ti,ab. | 17,594 |
| 16 | exp *health promotion/ | 45,186 |
| 17 | public health service/ | 75,674 |
| 18 | 8 or 9 or 10 or 11 or 12 or 13 or 14 or 15 or 16 or 17 | 950,845 |
| 19 | (evaluat* or decide* or decision* or case stud*).ti. | 1,204,304 |
| 20 | ("policy maker*" or policymaker* or "policy making" or policymaking).ti,kf. | 4,036 |
| 21 | (policy or policies).ab. /freq=3 | 55,268 |
| 22 | exp health care policy/ | 242,265 |
| 23 | 19 or 20 or 21 or 22 | 1,472,690 |
| 24 | exp population surveillance/ | 757 |
| 25 | infodemiology/ | 205 |
| 26 | (infodemiology or infoveillance).ti,kf. | 649 |
| 27 | (misinformation or disinformation).ti. | 2,127 |
| 28 | exp epidemic/pc [Prevention] | 9,936 |
| 29 | exp food poisoning/pc [Prevention] | 2,075 |
| 30 | ((prevent* or monitor* or control or surveillance or forecast* or predict* or model*) adj2 (outbreak* or pandemic* or epidemic*)).ti,kf. | 5,406 |
| 31 | exp *health care policy/ | 78,880 |
| 32 | 24 or 25 or 26 or 27 or 28 or 29 or 30 or 31 | 99,087 |
| 33 | 23 or 32 | 1,491,640 |
| 34 | 7 and 18 and 33 | 1,648 |
| 35 | limit 34 to yr="2020 -Current" | 1,154 |
| 36 | Public health.ti,kf. | 111,591 |
| 37 | *artificial intelligence/ or *large language model/ | 61,703 |
| 38 | (artificial intelligence or AI or llm* or machine learning or deep learning or expert system* or chatbot* or conversational agent*).ti,kf. | 224,025 |
| 39 | 37 or 38 | 236,453 |
| 40 | 39 and 36 | 929 |
| 41 | limit 40 to yr="2020 -Current" | 864 |
| 42 | 35 or 41 | 1,877 |

**Scopus**

| **#** | **Query** | **Results** |
| --- | --- | --- |
| 1 | ( TITLE ( "comput* intelligence" or "comput* reasoning" or "machine intelligence" or "artificial intelligence" or "natural language processing" or llm*1 or "large language model*" or "language learning model*" or "classification algorithm*" or "computer heuristic*" or "convolutional network*" or "expert system*" or "recommender system*" or "decision support system*" or "decision tree" or "deep learning" or "data science" or "feature detection" or "generative pre-trained transformer" or "generative pretrained transformer" or "learning algorithm*" or "machine learning" or "neural net*" or "reinforcement learning" or "*supervised learning" or "intelligent agent*" or "conversational agent*" or chatbot* or "Bing chat" or ChatGPT* or "Chat GPT" or "Google* Bard" or "Google* Gemini" or "IBM Watson" or "Microsoft* Bing" or Copilot or OpenAI or "Open AI" or PathAI or "Path AI" or DALL-E or invideo or Claude or Deepseek ) ) AND ( ( SRCTITLE ( public health ) ) OR ( TITLE ( "Public health" ) ) ) AND PUBYEAR > 2019 AND PUBYEAR < 2026 | 1,812 |

**Web of Science**

| **#** | **Query** | **Results** |
| --- | --- | --- |
| 1 | ( TITLE ( "comput* intelligence" or "comput* reasoning" or "machine intelligence" or "artificial intelligence" or "natural language processing" or llm*1 or "large language model*" or "language learning model*" or "classification algorithm*" or "computer heuristic*" or "convolutional network*" or "expert system*" or "recommender system*" or "decision support system*" or "decision tree" or "deep learning" or "data science" or "feature detection" or "generative pre-trained transformer" or "generative pretrained transformer" or "learning algorithm*" or "machine learning" or "neural net*" or "reinforcement learning" or "*supervised learning" or "intelligent agent*" or "conversational agent*" or chatbot* or "Bing chat" or ChatGPT* or "Chat GPT" or "Google* Bard" or "Google* Gemini" or "IBM Watson" or "Microsoft* Bing" or Copilot or OpenAI or "Open AI" or PathAI or "Path AI" or DALL-E or invideo or Claude or Deepseek ) ) AND ( ( SRCTITLE ( public health ) ) OR ( TITLE ( "Public health" ) ) ) AND PUBYEAR > 2019 AND PUBYEAR < 2026 | 732 |
